# Safety and Feasibility of Same Day Discharge Strategy for Primary Percutaneous Coronary Intervention

**DOI:** 10.5334/gh.1035

**Published:** 2021-07-01

**Authors:** Jehangir Ali Shah, Tahir Saghir, Bashir Ahmed, Syed Alishan ul Haq, Rajesh Kumar, Muhammad Naeem Mengal, Mehwish Zehra, Syeda Sakeena Raza, Musa Karim, Nadeem Qamar

**Affiliations:** 1 National Institute of Cardiovascular Diseases (NICVD), Karachi, PK; 2 Jinnah Post Graduate Medical Center (JPMC), Karachi, PK; 3 Jinnah Sindh Medical University (JSMU), Karachi, PK

**Keywords:** coronary artery disease, ST-segment elevation myocardial infarction, primary percutaneous coronary intervention, early discharge, major adverse cardiac events

## Abstract

**Background:**

The strategy for early discharge after primary percutaneous coronary intervention (PCI) could have substantial financial benefits, especially in low-middle income countries. However, there is a lack of local evidence on feasibility and safety of the strategy for early discharge. Therefore, the aim of this study was to assess the safety of early discharge after primary PCI in selected low-risk patients in the population of Karachi, Pakistan.

**Methods:**

In this study 600 consecutive low-risk patients who were discharged within 48 hours of primary PCI were put under observation for major adverse cardiac events (MACE) after 7 and 30 days of discharge respectively. Patients were further stratified into discharge groups of very early (≤ 24 hours) and early (24 to 48 hours).

**Results:**

The sample consisted of 81.8% (491) male patients with mean age of 54.89 ± 11.08 years. Killip class was I in 90% (540) of the patients. The majority of patients (84%) were discharged within 24 hours of the procedure. Loss to follow-up after rate at 7 and 30 days was 4% (24) and 4.3% (26) respectively. Cumulative MACE rate after 7 and 30 days was observed in 3.5% and 4.9%, all-cause mortality in 1.4% and 2.3%, cerebrovascular events in 0.9% and 1.4%, unplanned revascularization in 0.9% and 1.2%, re-infarction in 0.3% and 0.5%, unplanned re-hospitalization in 0.5% and 0.5%, and bleeding events in 0.5% and 0.5% of the patients respectively.

**Conclusion:**

It was observed that very early (≤ 24 hours) discharge after primary PCI for low-risk patients is a safe strategy subjected to careful pre-discharge risk assessment with minimal rate of MACE after 7-days as well as 30-days.

## Introduction

Atherosclerotic cardiovascular disease remains associated with leading rates of mortality and morbidity around the globe [1]. ST-segment elevation myocardial infarction (STEMI) remains the leading cause of visits to the cardiac emergency room, where the ultimate goal in the management of these patients is to restore myocardium blood flow. Hence, primary percutaneous coronary intervention (PCI) within 12 hours of onset of the symptoms remains the recommended reperfusion therapy [2,3]. Recent advancement in treatment modalities, adoption of guideline directed medical therapies, and evidence-based preventive measures have all contributed to improvement in prognosis of STEMI patients; however, a substantial number of patients still experience post-STEMI complications such as re-infarction, stent thrombosis, malignant arrhythmias, heart failure, and other mechanical complications [4,5,6,7,8]. This necessitates monitoring of these patients in the coronary care unit for at least 24 to 48 hours.

The management guidelines for STEMI developed by the European Society of Cardiology (ESC) recommends early discharge, within 72 hours, of patients at low risk of complications with adequate planning of follow-up and early rehabilitation [2]. Owing to the advancements in the management strategies and use of evidence based pharmacological therapies, there is a growing trend of reduced hospital stay for STEMI patients [9,10]. Feasibility, safety, and cost-effectiveness of the reduction of post-procedure hospital stay in selected low-risk STEMI patients has been demonstrated by some of the recent studies [11,12,13,14]. Randomized studies conducted thus far have established safety and feasibility of ≤ 72 hours post-procedure discharge strategy [11,14,15,16,17] and 48 to 56 hours post-procedure discharge strategy [18]. However, evidence from clinical trials with regards to next day discharge strategy (≤ 24 hour of procedure) is lacking; the only evidence of feasibility for next day discharge strategy from the Prague-5 trial is limited to a very limited number of patients [19].

Guidelines regarding management of STEMI patients is clear and well established; however, recommendations regarding appropriate time of discharge and identification of low-risk patients who may benefit the most from early discharge is not clear [18]. As a continuation of the effort to identify low-risk patients, the Zwolle Risk Score was developed, and has been recommended for clinical decision making of early discharge [20].

There is a lack of local evidence on the feasibility and safety of early discharge after primary PCI. The strategy of early discharge could not be more relevant in any healthcare system than that of a low-middle income country, similar to Pakistan. Appropriate and relevant evidence-based adoption of early discharge strategy in clinical practice could have a significant financial implication, from the perspective of both the patient and the hospital. Therefore, the aim of this study was to assess the safety of early discharge after primary PCI in selected low-risk patients at a tertiary care cardiac center in Karachi, Pakistan.

## Material and Methods

This prospective observational study was conducted at the largest cardiac care center of Pakistan, the National Institute of Cardiovascular Disease (NICVD), Karachi, Pakistan, from August 2019 to July 2020. Although the latter part of the study period coincided with the COVID-19 pandemic, with smart lockdowns imposed from 21^st^ March 2020 onwards, the emergency medical services remained without interruption during this period. The study was approved by the ethical review committee of the institution (ERC/38/2019). Successive patients presented to the emergency department with typical chest pain and diagnosed as STEMI according to the 4^th^ universal definition and undergone primary PCI within 12 hours of symptom onset were recruited for this study. Informed consent was obtained from all the patients with regards to participation in the study. Inclusion criteria for the study were STEMI patients of all genders, with ages between 18 to 80 years, Killip class I or II, and discharged within 48 hours of primary PCI. Patients who refused to participate in the study, or those with prior history of cardiac related surgery or intervention, or those who left against medical advice (LAMA), and ones with atrioventricular (AV) block were excluded from the study.

Patients’ baseline characteristics including demographic data and detailed history recording co-morbid conditions were collected and recorded on a structured proforma. All patients were managed as per practice guidelines and institutional protocols. All primary PCI procedures were performed by experienced cardiologists via transradial or transfemoral route; for procedures with transfemoral access the femoral sheath was removed by manual compression as per the institutional protocol and no vascular closure device was used. Angiographic findings, procedural details, and post-procedure complications (contrast induced nephropathy, bleeding (major bleeding as per TIMI bleeding criteria) [21], ventricular tachycardia, cardiac arrest, dissection, etc.) were recorded. Subsequent stage PCI on outpatient bases were planned for non-culprit vessels for all patients with multi-vessel coronary artery diseases. Zwolle risk score (ZRS) was calculated for all patients as per the scoring criteria defined by De Luca G et al [20]. Based on ZRS, patients were stratified into two groups; low-risk (≤ 3) and high-risk (≥ 4) respectively.

Decisions on early discharge were made by the treating physicians as per protocols of the institution. Given that the study’s center is a high-volume public-sector center that provides free-of-charge services with an average of 25 ± 5 primary PCI procedures per day, the institution protocols dictate to discharge low risk patients within 48 hour of procedure in order to maintain the patient to bed ratio.

All patients were prescribed with dual antiplatelet therapy, beta-blocker, angiotensin-converting enzyme inhibitor (ACEI)/angiotensin receptor blocker (ARB), and stains at discharge. Physical or telephonic follow-up after a week as well as after 30-days from procedure, respectively, was planned for all patients. Due to restrictions imposed by the COVID-19 pandemic, the majority of follow-ups were conducted via telephone. Outcomes were adjudicated at follow-up by the study investigator, either on physical examination or telephonic conversation (due to limited mobility caused by COVID-19 pandemic), with patients or legal caretaker. Follow-up outcomes included all-cause mortality, myocardial infarction, cerebrovascular event, unplanned hospitalization for unstable angina, and unplanned coronary revascularization.

Collected data was input and analyzed with the help of IBM SPSS version 21. Variables were expressed by computing appropriate summary statistics such as frequency, percentages, and mean ± standard deviations (SD) or median [interquartile range (IQR)]. Patients were stratified into two groups based on discharge time: Very early (≤ 24 hours) and early (24 to 48 hours), respectively. Baseline characteristics, angiographic findings, post procedure complications, and follow-up outcomes were compared for very early and early discharge groups, respectively, by applying the appropriate Student’s t-test or Mann–Whitney U test and Chi-square test, and a p-value ≤ 0.05 was considered statistically significant.

## Results

A total of 600 patients were included in the study with the proportion of males at 81.8% (491) and mean age of 54.89 ± 11.08 years. The median [IQR] duration of chest pain at presentation was 270 [150–450] minutes with majority (90%) of the patients in Killip class I. More than half (51.8%) of the patients presented with anterior wall MI followed by inferior wall MI (41.5%). Hypertension was the mostly commonly observed co-morbid condition, documented in 52.5% (315) of the patients, followed by diabetes (37.2%), and smoking (32.7%). Nearly half (47.5%) of the primary PCI procedures were performed through radial route. Post-procedure complication was observed in only 2% (12) of the patients, major bleeding was observed in only two patients–one with access site bleed and the other with gastrointestinal bleed. Details regarding complications are provided in Table 1.

**Table 1:** Baseline characteristics, angiographic findings, procedural characteristics, and post procedure complications stratified by discharge time.

Characteristics	Total	Discharge after Primary PCI		P-value

		Very Early(≤ 24 hours)	Early(24 to 48 hours)	

**Total (N)**	**600**	**504**	**96**	**–**
**Gender**				
Male	81.8% (491)	83.1% (419)	75% (72)	0.058
Female	18.2% (109)	16.9% (85)	25% (24)	
**Age (years)**	54.89 ± 11.08	54.63 ± 10.84	56.23 ± 12.23	0.195
≤ 45 years	21% (126)	21.2% (107)	19.8% (19)	0.230
46 to 65 years	63% (378)	63.9% (322)	58.3% (56)	
> 65 years	16% (96)	14.9% (75)	21.9% (21)	
**Killip class**				
I	90% (540)	91.5% (461)	82.3% (79)	0.006*
II	10% (60)	8.5% (43)	17.7% (17)	
**Duration of CP (minutes)**	270 [150–450]	270 [150–448]	300 [145–570]	0.311
**Type of myocardial infarction**				
Anterior	51.8% (311)	53% (267)	45.8% (44)	0.435
Inferior	41.5% (249)	40.5% (204)	46.9% (45)	
Posterior	4.7% (28)	4.4% (22)	6.3% (6)	
Lateral	2% (12)	2.2% (11)	1% (1)	
**Co-morbid conditions**				
Hypertension	52.5% (315)	50.4% (254)	63.5% (61)	0.018*
Diabetes	37.2% (223)	36.7% (185)	39.6% (38)	0.593
Family history of CAD	4.5% (27)	4.6% (23)	4.2% (4)	0.864
Smoking	32.7% (196)	34.3% (173)	24% (23)	0.047*
Obesity	4.3% (26)	4.6% (23)	3.1% (3)	0.526
**Access for the procedure**				
Radial	47.5% (285)	50% (252)	34.4% (33)	0.005*
Femoral	52.5% (315)	50% (252)	65.6% (63)	
**Number of vessels involved**				
Single vessel disease	22.8% (137)	23.6% (119)	18.8% (18)	0.066
Two vessels disease	36.8% (221)	38.1% (192)	30.2% (29)	
Three vessels disease	40.3% (242)	38.3% (193)	51% (49)	
**Culprit coronary artery**				
Left main	1.7% (10)	1.8% (9)	1% (1)	0.513
LAD	55.7% (334)	56.9% (287)	49% (47)	
RCA	29.5% (177)	28% (141)	37.5% (36)	
LCX	11.5% (69)	11.5% (58)	11.5% (11)	
Ramus	1.2% (7)	1.2% (6)	1% (1)	
Diagonal	0.5% (3)	0.6% (3)	0% (0)	
**TIMI (pre-procedure) flow grade**				
0	52.2% (313)	49.4% (249)	66.7% (64)	0.010*
I	40.7% (244)	42.5% (214)	31.3% (30)	
II	4.5% (27)	5.2% (26)	1% (1)	
III	2.7% (16)	3% (15)	1% (1)	
**TIMI (post-procedure) flow grade**				
0	1% (6)	1% (5)	1.1% (1)	<0.001*
I	0.5% (3)	0.2% (1)	2.1% (2)	
II	17.5% (103)	14.3% (71)	34% (32)	
III	81% (477)	84.4% (418)	62.8% (59)	
**Complication(s)/outcomes**				
**Total**	2% (12)	0.8% (4)	8.3% (8)	<0.001*
Contrast-induced nephropathy (CIN)	16.7% (2)	0% (0)	25% (2)	0.001*
Major bleeding	16.7% (2)	25% (1)	12.5% (1)	0.189
Ventricular tachycardia	16.7% (2)	25% (1)	12.5% (1)	0.189
Cardiac arrest	16.7% (2)	0% (0)	25% (2)	0.001*
Dissection	16.7% (2)	25% (1)	12.5% (1)	0.189
Others	16.7% (2)	25% (1)	12.5% (1)	0.189

* Significant at 5%. PCI = percutaneous coronary intervention; CP = chest pain; CAD = coronary artery diseases; LAD = left anterior descending artery; RCA = right coronary artery; LCX = left circumflex artery; TIMI = thrombolysis in myocardial infarction.

The majority of patients (84%) were discharged within 24 hours of the procedure and the remaining 16% were discharged after 24 to 48 hours of primary PCI. Demographic and clinical characteristics of very early (≤ 24 hours) and early (24 to 48 hours) discharge group of patients were mostly similar, except that the patients in very early discharge groups had significantly higher proportion of Killip class I, lower proportion of hypertension, higher number of smokers, lesser number of procedures through femoral access, better pre- and post-procedure TIMI flow grade, and lesser post-procedure complications (Table 1).

Follow-up after 7 days was successfully completed for 576 patients with loss to follow-up rate of 4% (24). Major adverse cardiac event (MACE) was observed for only 3.5% (20) of the cases with all-cause mortality rate of 1.4% (8), cerebrovascular events rate of 0.9% (5), myocardial infarction rate of 0.3% (2), unplanned coronary revascularization rate of 0.9% (5), unplanned hospitalization for unstable angina rate of 0.5% (3), and bleeding events were reported by 0.5% (3) of the patients. MACE rate after 7 days of procedure was not statistically different for very early and early discharge groups.

Similarly, follow-up after 30 days was successfully completed for 574 patients with loss to follow-up rate of 4.3% (26). Cumulative MACE rate after 30 days was observed to be 4.9% (28) with 2.3% (13) all-cause mortality, 1.4% (8) cerebrovascular events, 1.2% (7) unplanned coronary revascularization, 0.5% (3) unplanned hospitalization for unstable angina, and 0.3% (2) bleeding events. MACE rate after 30 days of procedure was also not statistically different for very early and early discharge groups. Post-procedure 7-day and 30-day follow-up outcomes for early discharged patients stratified by discharge time are presented in Table 2.

**Table 2:** Post-procedure 7-day and 30-day follow-up outcomes stratified by discharge time.

Outcomes	Total	Discharge after Primary PCI		P-value

		Very Early (≤ 24 hours)	Early (24 to 48 hours)	

**Total (N)**	**600**	**504**	**96**	**–**
**Outcomes after 7-days**				

**Loss to follow-up**	**4% (24)**	**3% (15)**	**9.4% (9)**	–
**Total (N) at 7-day follow-up**	**576**	**489**	**87**	–
MACE	3.5% (20)	3.7% (18)	2.3% (2)	0.516
All-cause death	1.4% (8)	1.6% (8)	0% (0)	0.230
Myocardial infarction	0.3% (2)	0.4% (2)	0% (0)	0.550
Bleeding events	0.5% (3)	0.6% (3)	0% (0)	0.464
Cerebrovascular events	0.9% (5)	0.8% (4)	1.1% (1)	0.759
Hospitalization for unstable angina	0.5% (3)	0.4% (2)	1.1% (1)	0.377
Unplanned coronary revascularization	0.9% (5)	1% (5)	0% (0)	0.343
**Outcomes after 30 days**				

**Loss to follow-up**	**4.3% (26)**	**3.4% (17)**	**9.4% (9)**	–
**Total (N) at 30-day follow-up**	**574**	**487**	**87**	–
MACE	4.9% (28)	5.1% (25)	3.4% (3)	0.501
All-cause death	2.3% (13)	2.5% (12)	1.1% (1)	0.448
Myocardial infarction	0.5% (3)	0.6% (3)	0% (0)	0.463
Bleeding events	0.5% (3)	0.6% (3)	0% (0)	0.463
Cerebrovascular events	1.4% (8)	1.4% (7)	1.1% (1)	0.833
Hospitalization for unstable angina	0.5% (3)	0.4% (2)	1.1% (1)	0.379
Unplanned coronary revascularization	1.2% (7)	1.4% (7)	0% (0)	0.261

* Significant at 5%. PCI = percutaneous coronary intervention; MACE = major adverse cardiac event.

Outcomes after 7 and 30 days of procedure were not significantly different for patients in low- and high-risk groups. MACE and all-cause 30-day mortality were found to be relatively higher among high-risk group with MACE and mortality rate of 5.8% vs. 4.7%; p = 0.641 and 3.8% vs. 1.9%; p = 0.231 respectively. Post-procedure 7- and 30-day follow-up outcomes for early discharged patients stratified based on Zwolle risk score (ZRS) are presented in Table 3.

**Table 3:** Post-procedure 7-day and 30-day follow-up outcomes stratified by Zwolle risk score (ZRS).

Outcomes	Total	Zwolle risk score (ZRS)		p-value

		Low-risk (≤ 3)	High-risk (≥ 4)	

**Total (N)**	**600**	**491**	**109**	**–**
**Outcomes after 7-days**				

**Loss to follow-up**	**4% (24)**	**3.9% (19)**	**4.6% (5)**	–
**Total (N) at 7-day follow-up**	**576**	**472**	**104**	–
MACE	3.5% (20)	3.6% (17)	2.9% (3)	0.718
All-cause death	1.4% (8)	1.5% (7)	1% (1)	0.681
Myocardial infarction	0.3% (2)	0.4% (2)	0% (0)	0.506
Bleeding events	0.5% (3)	0.6% (3)	0% (0)	0.415
Cerebrovascular events	0.9% (5)	0.8% (4)	1% (1)	0.910
Hospitalization for unstable angina	0.5% (3)	0.6% (3)	0% (0)	0.415
Unplanned coronary revascularization	0.9% (5)	0.8% (4)	1% (1)	0.910
**Outcomes after 30 days**				

**Loss to follow-up**	**4.3% (26)**	**4.3% (21)**	**4.6% (5)**	–
**Total (N) at 30-day follow-up**	**574**	**470**	**104**	–
MACE	4.9% (28)	4.7% (22)	5.8% (6)	0.641
All-cause death	2.3% (13)	1.9% (9)	3.8% (4)	0.231
Myocardial infarction	0.5% (3)	0.4% (2)	1% (1)	0.493
Bleeding events	0.3% (2)	0.4% (2)	0% (0)	0.505
Cerebrovascular events	1.4% (8)	1.5% (7)	1% (1)	0.678
Hospitalization for unstable angina	0.5% (3)	0.6% (3)	0% (0)	0.414
Unplanned coronary revascularization	1.2% (7)	1.3% (6)	1% (1)	0.791

MACE = major adverse cardiac event.

## Discussion

The strategy of early discharge after primary PCI for STEMI patients has been repeatedly studied in recent years. The aim of this study was to assess the safety of the early discharge strategy in selected low-risk patients at a tertiary care cardiac center in Karachi, Pakistan. We observed major adverse cardiac events after 7 days and 30 days in 600 early discharged low-risk patients respectively. More than 80% of the patients were discharged from hospital within 24 hours of primary PCI. Cumulative MACE rate after 7 days and 30 days was observed to be 3.5% and 4.9% respectively. All-cause mortality rate was 1.4% and 2.3%; cerebrovascular events rate was 0.9% and 1.4%; unplanned revascularization rate was 0.9% and 1.2%; re-infarction rate was 0.3% and 0.5%; unplanned re-hospitalization rate was 0.5% and 0.5%; and bleeding events rate was 0.5% and 0.5% for patients after 7 days and 30 days respectively.

A recent meta-analysis of five randomized control trials comprised of 1575 STEMI patients on safety of early discharge after primary PCI by Gong W et al [22]. reported there to be no difference in 30-days mortality and readmission rates with risk ratio (RR) of 0.65 [0.38–1.12] and 1.18 [0.52–2.69] respectively between early discharged (≤ 72 hours) and conventionally discharged patients. This meta-analysis concluded that the early discharge strategy was safe and could benefit both the healthcare system and patients. Melberg T et al [15]. conducted a randomized control trial including 425 surviving primary PCI low-risk (Zwolle risk score ≤ 3) patients, randomized to either usual discharge or early discharge (≤ 72 hours), and reported no increase in 30-days readmission rate (2.8% vs. 3.7%; p = 0.690 respectively) with no difference in health status measurements. Another randomized study by Satılmısoglu MH et al [18]. tested safety hypothesis of discharge within 48 to 56 hours as opposed to usual discharge, and reported no difference in all-cause mortality (0.6% vs. 0.8%; p = 0.369) and readmission (3.8% vs. 6.9%; p = 0.061) rate after 30-days of discharge respectively. Similarly, many other studies conducted in various parts of the world showed evidence in favor of the early discharge (≤ 72 hours) strategy [11,12,13,14,23].

However, evidence regarding safety of same day (≤ 24 hours) discharge strategy in setting of primary PCI is very limited as safety of this strategy after successful ad hoc or elective PCI has been reported by only a few studies [24,25,26]. In this study very early (≤ 24 hours) discharge after primary PCI has been shown to be feasible and safe, subject to the careful risk assessment of patients. Such a strategy can have financial and logistical benefits for both patients as well as the healthcare systems. The average cost of an additional day of hospital care after primary PCI can be up to PKR 25,000 and may also result in increased bed occupancy in a busy center such as that used in this study. The commonly adopted risk stratification approaches for early discharge include Zwolle risk score (ZRS) ≤ 3 [15,16], CADILLAC risk score ≤ 2 [27], and NT-proBNP (< 200 pg/mL) or ZRS (< 2) [8].

To the best of the authors’ knowledge, this is the first study from this region on the assessment of very early discharge strategy in primary PCI settings; however, major limitations of the study include nonrandomized study design, lack of comparative normal discharge group, and single center coverage with a relatively small sample size.

## Conclusion

Very early (≤ 24 hours) discharge after primary PCI for low-risk patients is a safe strategy subject for careful pre-discharge risk assessment with minimal rate of MACE after 7-days as well as 30-days. Adoption of early discharge strategy in clinical practice at a larger scale could have substantial financial benefits for patients as well as healthcare systems of low-middle income economies.

## Data Accessibility Statement

Data of the study will be available upon request.
